# Geographical variation in compulsory mental health care: cause for concern and source of causal inference

**DOI:** 10.1192/j.eurpsy.2024.161

**Published:** 2024-08-27

**Authors:** T. Hofstad

**Affiliations:** Haukeland University Hospital, Bergen, Norway

## Abstract

**Introduction:**

Compulsory mental health care remains a controversial practice. The many difficulties in performing Randomised Controlled Trials (RCT) on the topic means there is limited evidence to support its effectiveness. For ethical and legal reasons, compulsory mental health care should only be used when necessary. Yet, geographical variations, which can indicate both overuse and underuse, have been observed. In the funded research project “Controversies in Psychiatry” we intend to use this variation as a source of knowledge production. We propose that this naturally occurring variation mimics randomisation, and can therefore permit causal inference from registry data.

**Objectives:**

We will estimate the causal effect of compulsory inpatient mental health care on a range of outcomes, including injuries, self-harm, and all-cause mortality; violent crime; employment vs benefit allowance; rehospitalisation and outpatient commitment.

**Methods:**

Observed variation in register data on all episodes of compulsory inpatient mental health care in Norway between 2015-2016 (N ≈ 300 000), will serve as a source of as-random variation. Provider-preference for compulsion usage will be used as an instrumental variable (IV).

**Results:**

Outcomes will be observed from 2017-2025. If assumptions underlying IV-analysis do not hold, the project will still provide important and complete descriptive data on long-term outcomes for a whole population.

**Conclusions:**

Geographical variation is a cause for concern if people are treated differently depending on area of residence. But it also presents an opportunity to use differences in service provider’s preference for using compulsory care as an instrumental variable to estimate the causal effect of compulsory care on multiple short and long-term outcomes. This approach can help resolve controversies that are difficult or even impossible to investigate through RCTs. After presenting the project plan we invite to a discussion of the feasibility of using an instrument variable approach to explore if relatively low versus high rates of compulsory care produce favorable outcomes for patients.

**Disclosure of Interest:**

None Declared

